# Novel Reassortant Avian Influenza A(H5N6) Virus, China, 2021

**DOI:** 10.3201/eid2808.212241

**Published:** 2022-08

**Authors:** Junhong Chen, Lingyu Xu, Tengfei Liu, Shumin Xie, Ke Li, Xiao Li, Mengmeng Zhang, Yifan Wu, Xinkai Wang, Jinfeng Wang, Keyi Shi, Beibei Niu, Ming Liao, Weixin Jia

**Affiliations:** South China Agricultural University College of Veterinary Medicine, Guangzhou, China (J. Chen, L. Xu, T. Liu, S. Xie, X. Li, M. Zhang, Y. Wu, X. Wang, J. Wang, K. Shi, B. Niu, M. Liao, W. Jia);; Experimental Animal Center, South China Agricultural University, Guangzhou (S. Xie);; Yunnan Animal Science and Veterinary Institute, Kunming, Yunnan, China (K. Li);; Key Laboratory of Zoonoses and Key Laboratory of Animal Vaccine Development, Ministry of Agriculture, Guangzhou, China (M. Liao, W. Jia);; Key Laboratory of Zoonoses Prevention and Control of Guangdong Province, Guangzhou (W. Jia)

**Keywords:** influenza, poultry diseases, reassortant viruses, influenza A virus, avian influenza A(H5N6) virus, viral zoonoses, zoonoses, China, viruses

## Abstract

Although reports of human infection with influenza A(H5N6) increased in 2021, reports of similar H5N6 virus infection in poultry are few. We detected 10 avian influenza A(H5N6) clade 2.3.4.4b viruses in poultry from 4 provinces in China. The viruses showed strong immune-escape capacity and complex genetic reassortment, suggesting further transmission risk.

Severe human infection with influenza A(H5N6) virus was identified in China in 2014. During 2014–2020, a total of 26 cases of human infection were laboratory confirmed (*1*,*2*). Sporadic cases did not attract widespread attention until 2021 (*3*,*4*). During February–October 2021, China reported 24 laboratory-confirmed cases of human infection with H5N6 virus and 5 deaths (Figure 1, panel A); the number of human infections within only 8 months was close to the total for the previous 7 years.

**Figure 1:**
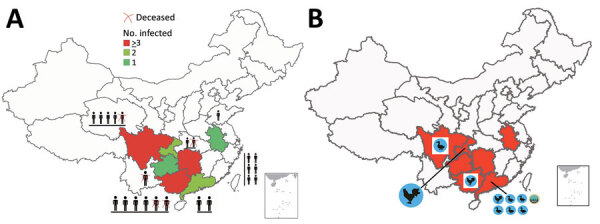
Distribution of confirmed cases of avian influenza A(H5N6) virus in humans, China, 2021. A) Provinces of the outbreaks and number of infected persons. A red X indicates a deceased person, and colors represent the number of infected persons. B) Region of novel H5N6 virus isolation from birds (chickens, ducks) and the environment (green icon). The red areas on the map indicate the provinces where human cases occurred in 2021. Insets indicate islands of China, additional sites of poultry breeding and human habitation.

The policy of compulsory poultry immunizations in China was adopted to prevent and control infection with highly pathogenic avian influenza (HPAI) subtype H5Nx (*5*). Although vaccination can reduce the likelihood of severe clinical disease and reduce shedding of virus in poultry, it cannot prevent sporadic infections with H5N6 virus in waterfowl. Because it is difficult to achieve a qualified 100% rate of H5N6 virus antibodies in waterfowl (*6*), these birds have become a weak link in prevention and control of the virus. In the context of selection pressure for vaccines and the absence of immunity in waterfowl, antigenic drift causes the H5N6 virus to continuously evolve (*7*), making currently available H5N6 vaccines ineffective.

On November 27, 2020, an outbreak of influenza A(H5N8) virus infections among wild swans was reported in China, resulting in the death of 2 swans (*8*,*9*). Since then, H5N8 clade 2.3.4.4b virus has spread throughout China, resulting in co-endemicity of H5N6 clade 2.3.4.4h/b and H5N8 clade 2.3.4.4b viruses. This 2020 outbreak was not the first outbreak of H5N8 virus in China; the earliest introduction of the virus into China was reported in Liaoning on September 12, 2014 (*10*,*11*). Because of China’s immunization policies for poultry, H5N8 virus was quickly eliminated, only to reemerge in 2020. 

The reappearance and spread of H5N8 virus is a serious threat to the poultry industry. The ecologic environment of the virus has been altered, given the increasing number of influenza A(H5N6) cases in humans. The current prevalence and mode of virus reassortment is of great concern. We discovered a novel H5N6 virus that has spread throughout the poultry industry, and similar viruses caused a sharp rise in human infections.

## The Study

In June 2021, we isolated an H5N6 virus from a sick duck on a live poultry farm in Chengdu, Sichuan, China. In July 2021, we isolated another H5N6 virus from a dead chicken in the backyard of a human patient with confirmed infection in Chongqing. In August 2021, we detected an H5N6 virus on a chicken farm in Maoming City and another on a goose farm in Huizhou City (both cities in Guangdong, China). In September 2021, we detected an H5N6 virus on a chicken farm in Qinzhou, Guangxi. Last, in October 2021, we detected 5 H5N6 viruses at live poultry markets in Dongguan City of Guangdong Province (3 from ducks, 1 from a goose, and 1 from the environment) (Figure 1, panel B).

**Figure 2:**
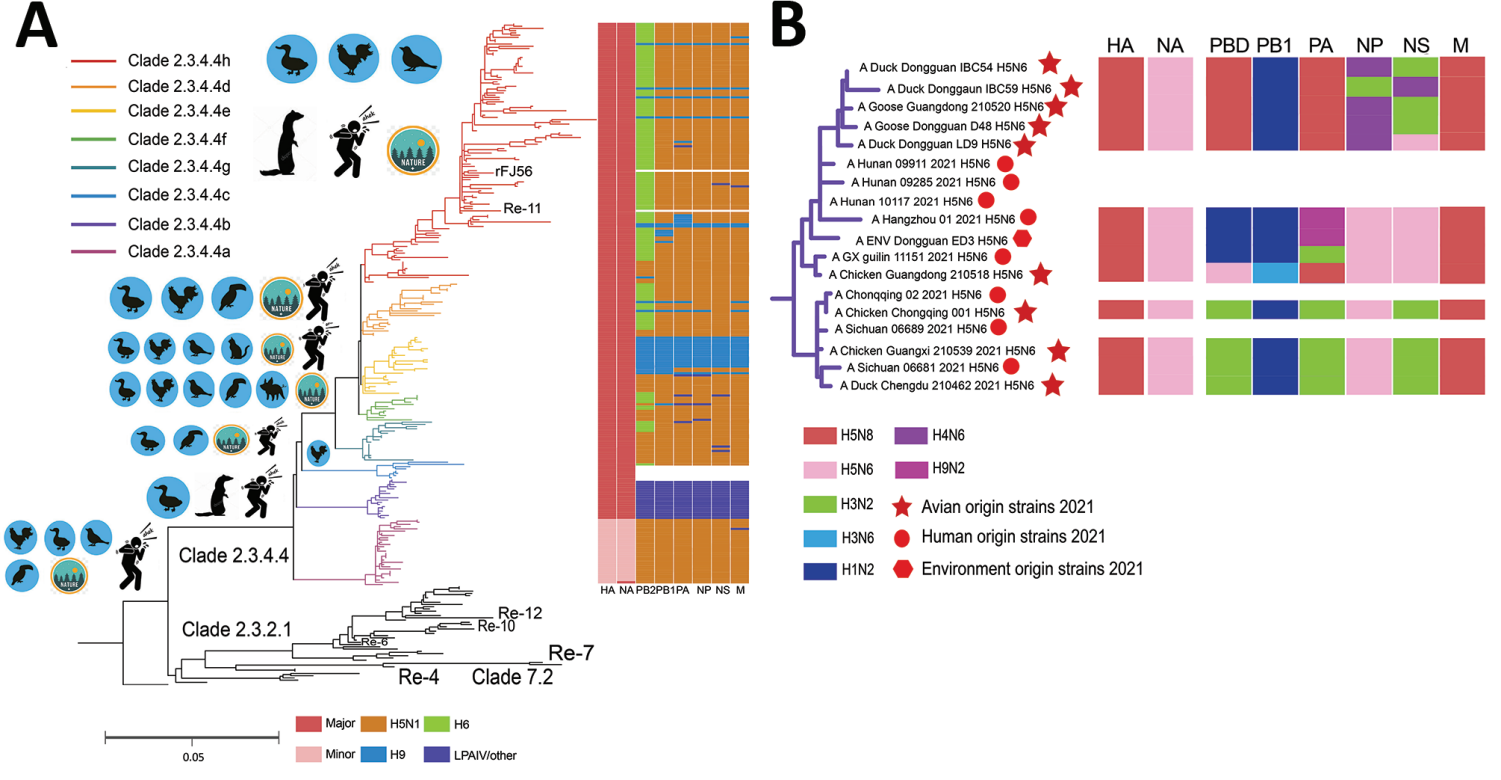
Visual depictions of avian influenza A(H5N6) viruses from China, 2021, and reference viruses. A) Maximum-likelihood phylogenetic tree showing comparisons with 332 H5 reference sequences downloaded from the GISAID database (http://www.gisaid.org). The Guizhou strain (A/Guizhou/1/2012) was set as the tree root, and all influenza A(H5N1) strains were set as the outgroup. Re-X/rFJ56 represents vaccine strains. To the left of each clade are images showing the corresponding primary hosts. On the right side is the dynamic reassortment profile of each avian (H5N6 virus in the phylogenetic tree; colors represent gene segments. Colored boxes below the graph correspond to possible potential donor viruses. B) Novel avian and environmental origin H5N6 strains. Red circles represent human strains (Appendix Tables 13–17). HA, hemagglutinin; LPAIV, low-pathogenicity avian influenza virus; NA, neuraminidase; NS, nonstructural; M, matrix. PA, polymerase acidic; PB, polymerase basic.

To examine the genetic relationships of the viruses, we sequenced the genomes of the 10 H5N6 viruses and constructed maximum-likelihood phylogenetic trees divided into H5N6 epidemic clades (*12*), according to the protocol established by the World Health Organization (Figure 2, panel A). The H5N6 virus fell within 8 hemagglutinin (HA) clades (2.3.4.4a to 2.3.4.4h). The similarity between the HA genes of all 10 viruses was 99.1%–100%, and all belonged to clade 2.3.4.4b. The HA genes of all the strains had the typical HPAI virus amino acid sequence RRKR↓GLF at the cleavage site (Appendix Table 1). All viruses contain the S137A and T192I mutations in the HA gene, which enable it to bind to the human α-2, 6-linked sialic acid receptor, thereby increasing human susceptibility to the virus (*8*,*13*). Mammalian adaptive mutations, such as T33K, L89V, and G309D (*14*), were detected in the polymerase basic (PB) 2 gene of all strains, which increases the virulence of H5 viruses in mice (Table 1). Those variants are uncommon in previously circulating H5N6 (clade 2.3.4.4h) viruses.

**Table 1:** Mutation sites for novel influenza A(H5N6) avian influenza viruses detected from humans and birds, China, 2021*

Strain	HA gene	Function	PB2 gene	Function	Host
A/Chongqing/00013/2021/H5N6	S137A/T192I	Increased α-2,6 sialic acid receptor affinity	T33K/L89V/G309D	Enhanced virulence of H5 viruses in mice	Human
A/Sichuan/06681/2021/H5N6					Human
A/Sichuan/06689/2021/H5N6					Human
A/Hunan/09285/2021/H5N6					Human
A/Hunan/09911/2021/H5N6					Human
A/Chongqing/02/2021/H5N6					Human
A/chicken/Chongqing/001/2021/H5N6V†					Avian
A/goose/Guangdong/210520/2021/H5N6V†					Avian
A/chicken/Guangdong/210518/2021/H5N6†					Avian
A/duck/Chengdu/210462/2021/H5N6†					Avian
A/goose/Dongguan/D48/2021/H5N6†					Avian
A/duck/Dongguan/LD9/2021/H5N6†					Avian
A/ENV/Dongguan/ED3/2021/H5N6†					Environment
A/duck/Dongguan/IBC54/2021/H5N6†					Avian
A/duck/Dongguan/IBC59/2021/H5N6†					Avian
A/chicken/Guangxi/210539/2021/H5N6†					Avian
A/whooper swan/Xinjiang/13/2020/H5N6‡	137S/192T	None			Avian
A/chicken/Suzhou/j6/2019/H5N6‡	137S/192T	None			Avian
A/China/Original/2018/H5N6‡	137S/192T	None			Human

*HA, hemagglutinin; PB2, polymerase basic 2. †Avian and environmental strains isolated in study of novel reassortant avian influenza A(H5N6) virus, China, in 2021. The remaining reference strains were downloaded from GISAID (http://www.gisaid.org). All strains isolated from humans in 2021 were novel A(H5N6) viruses. ‡Reference strain (clade 2.3.4.4h) that did not have HA gene mutation before 2021.

Because there was an epidemiologic correlation between the avian virus strains from Chongqing and the human infections in Chongqing, we used Chongqing avian strains as a representative virus for the control analysis with human-origin virus sequences (Table 2). We deemed this approach to be the appropriate way to reveal links between the human-origin and avian-origin viruses. We found many similarities between the PB2, polymerase acidic, and nonstructural genes of the Chongqing avian-origin strain and those in influenza A(H3N2) virus, which suggests that the novel virus reassorted with H3N2 virus. The PB1 gene is derived from influenza A(H1N2) virus. The HA gene of the Chongqing H5N6 virus was 99.2% similar to that of the H5N8 virus and the matrix genes were 99.9% similar to those of the H5N8 virus, which leads us to believe that both genes originated from the H5N8 virus. The neuraminidase gene was derived from the H5N6 virus, however, and we speculate that the novel H5N6 virus is a reassortment of the H5N8 and H5N6 viruses. The HA and matrix genes of all viruses were derived from H5N8, which suggests that the novel H5N6 virus may use the gene backbone of the H5N8 virus. Other low-pathogenicity avian influenza viruses have been found to be involved in reassortment, which makes the internal genes of all strains appear complex but inconsistent (Appendix Tables 2–11). Notably, >1 pattern of reassortment seems to be present. Some human-derived strains have internal genes that are close to known H5N6 HPAI virus genes, and others are less closely related (Figure 2, panel B).

**Table 2:** Genomic similarity of influenza virus isolate A/chicken/Chongqing/001/2021/H5N6 from a bird in China, 2021, to previously detected influenza viruses from birds in China*

Gene	Name	Subtype	Similarity, %	Host	Year
PB2	A/chicken/Guangxi/165C7/2014(H3N2)	H3N2	96.90	Chicken	2014
PB1	A/duck/Guangxi/293D21/2017(H1N2)	H1N2	97.90	Duck	2017
PA	A/duck/China/322D22/2018(H3N2)	H3N2	96.51	Duck	2018
NP	A/Muscovy duck/China/H5N6/2020(H5N6)	H5N6	95.20	Duck	2020
NS	A/chicken/Ganzhou/GZ43/2016(H3N2)	H3N2	97.90	Chicken	2016
M	A/Cygnus columbianus/Hubei/56/2020(H5N8)	H5N8	99.90	Cygnus	2020
HA	A/Cygnus columbianus/Hubei/53/2020(H5N8)	H5N8	99.20	Cygnus	2020
NA	A/Muscovy duck/China/H5N6/2020(H5N6)	H5N6	99.28	Duck	2020

*The virus isolate came from a dead chicken in the backyard of a patient with confirmed infection in Chongqing, China. The host, subtype, and similarity of reference sequences were obtained from the National Center for Biotechnology Information (https://www.ncbi.nlm.nih.gov). HA, hemagglutinin; NA, neuraminidase; NS, nonstructural; M, matrix. PA, polymerase acidic; PB, polymerase basic.

Bayesian analysis indicated that the viruses in the Pearl River Delta region (Guangdong), the upper reaches of the Yangtze River (Sichuan/Chongqin), and the middle and lower reaches of the Yangtze River (Hunan/Hangzhou) formed 3 subclades according to geographic characteristics (Appendix Figure 1). Compared with the study of Gu et al. (*15*), we found that the domestic novel H5N6 virus initially formed 3 subclades and an additional 7 types of genomes (Figure 2, panel B). Current vaccine strains lack protection (hemagglutination inhibition test) against novel H5N6 circulating virus strains (Appendix Table 12). This result differs from that reported by Gu et al. (*15*), which may result from isolation of the virus from different regions. Our study suggests that the virus poses a high risk for further transmission, which can be reduced or avoided by updating the vaccine strains. In January 2022, the Chinese government introduced new vaccine strains H5-Re14 and rHN5801 to prevent an epidemic of poultry infection with H5N6 virus.

Because reported cases in humans were concentrated in southern China within a short time and most of these cases happened during the noninfluenza season, we suspect that transmissibility or viral load of the novel H5N6 viruses has increased. Furthermore, the virus that was isolated from environmental swab specimens (sewage ditch swab in a live poultry market) in Dongguan indicates that the virus is already present in the surrounding environment, which could increase the likelihood that the virus will infect humans.

## Conclusions

At the peak of human cases, we isolated a total of 10 novel reassortment H5N6 virus strains from local poultry and the environment that were highly similar to the H5N6 (human-origin) virus reported during the same period. The human and avian viruses belong to clade 2.3.4.4b. The initial epidemic strains clustered into 3 geographically characterized subclades, and each avian strain had the same mammalian susceptibility mutation. The apparent antigenic differences between the virus and vaccine antiserum suggest further transmission risk.

Appendix Supplemental methods and results for study of novel reassortant avian influenza A(H5N6) virus, China, 2021.
