# Supplementary material for: Novel Reassortant Avian Influenza A(H5N6) Virus, China, 2021
Source: Emerg Infect Dis. 2022 Aug;28(8):1703–7. doi: 10.3201/eid2808.212241 (PMC9328904; doi:10.3201/eid2808.212241)
Supplement: Appendix — Supplemental methods and results for study of novel reassortant avian influenza A(H5N6) virus, China, 2021. [file 21-2241-Techapp-s1.pdf]

Article DOI: https://doi.org/10.3201/eid2808.212241

# Novel Reassortant Avian Influenza A(H5N6) Virus, China, 2021

## Appendix

## Methods

### Sample collection and virus isolation

For the farm poultry, we collected tissues from the organs, larynxes, and lungs, and we collected throat and cloacal swabs for the birds in the LPM. We isolated the viruses in SPF chicken embryos and conducted RT-PCR amplification and sequencing. Throat and cloacal swab samples of poultry from live poultry markets were collected in Guangdong and Guangxi provinces, China. Disease tissues were from Chongqing and Chengdu provinces of China, where Chongqing disease samples were from dead chickens at the rear of confirmed cases. Each sample was placed in 2 ml of the PBS supplemented with penicillin (5000 U/ml) and streptomycin (5000 U/ml). All the samples were inoculated in the allantoic cavities of 10-day-old SPF embryonated chicken egg at 37°C. The allantoic fluid was collected and tested for hemagglutinin (HA) assay with 1% chicken red blood cells and then used in this study.

### RNA extraction, RT-PCR, and DNA sequencing

RNA was extracted from the suspension of virus isolates with the RNeasy Mini Kit (Fastagen) as directed by the manufacturer. Two-step RT-PCR was conducted with universal primers as previously described (*1*), and the genome of H5N6 was amplified under standard conditions. PCR products were purified with a QIAamp Gel extraction kit (Qiagen) and sequenced with an ABI 3730 DNA Analyzer (Applied Biosystems).

**Phylogenic analysis**

All the available genomic sequences with the complete coding regions of influenza A(H5N6) viruses were downloaded from GISAID (http://www.gisaid.org/). The genome of H5N6 sequences data set (sequences alignment was available on request) was then created. The downloaded genomic sequences together with new 10 H5N6 strains were aligned using the MAFFT (version 7.149) program (*2*). The ML (Maximum likelihood) phylogenetic tree of each gene fragment was constructed by Phylosuite software (v 1.2.2) (*3*). Number of bootstrap with 5000 and Modle with GTR. The phylogenetic tree was visualized in the iTOL (version 6) website (https://itol.embl.de/).

**Statistical analyses**

Fluctuation curves of photoreceptors were generated by GraphPad Prism 5.0.

**References**


1. Bi Y, Chen Q, Wang Q, Chen J, Jin T, Wong G, et al. Genesis, Evolution and Prevalence of H5N6 Avian Influenza Viruses in China. Cell Host Microbe. 2016;20:810–21. PubMed https://doi.org/10.1016/j.chom.2016.10.022

2. Katoh K, Misawa K, Kuma K, Miyata T. MAFFT: a novel method for rapid multiple sequence alignment based on fast Fourier transform. Nucleic Acids Res. 2002;30:3059–66. PubMed https://doi.org/10.1093/nar/gkf436

3. Zhang D, Gao F, Jakovlić I, Zou H, Zhang J, Li WX, et al. PhyloSuite: An integrated and scalable desktop platform for streamlined molecular sequence data management and evolutionary phylogenetics studies. Mol Ecol Resour. 2020;20:348–55. PubMed https://doi.org/10.1111/1755-0998.13096

**Appendix Table 1.** Information on the influenza A(H5N6) viruses in this study

| Name | Location | Host | Date | Subtype | Cleavage site sequence |
|---|---|---|---|---|---|
| A_Duck_Chengdu_210462_2021_H5N6 | Chengdu | Duck | 2021/6/30 | H5N6 | RRKR↓GLF |
| A_Chicken_Chongqing_001_2021_H5N6 | Chongqing | Chicken | 2021/7/29 | H5N6 | RRKR↓GLF |
| A_Goose_Guangdong_210520_2021_H5N6 | Guangxi | Chicken | 2021/8/24 | H5N6 | RRKR↓GLF |
| A_Chicken_Guangdong_210518_2021_H5N6 | Guangdong | Chicken | 2021/8/24 | H5N6 | RRKR↓GLF |
| A_Chicken_Guangxi_210539_2021_H5N6 | Guangdong | Chicken | 2021/9/23 | H5N6 | RRKR↓GLF |
| A_Goose_Dongguan_D48_2021_H5N6 | Guangdong | Chicken | 2021/9/2 | H5N6 | RRKR↓GLF |
| A_Duck_Dongguan_LD9_2021_H5N6 | Guangdong | Chicken | 2021/9/15 | H5N6 | RRKR↓GLF |
| A_ENV_Dongguan_ED3_2021_H5N6 | Guangdong | Chicken | 2021/9/1 | H5N6 | RRKR↓GLF |
| A_Duck_Dongguan_IBC54_2021_H5N6 | Guangdong | Chicken | 2021/9/7 | H5N6 | RRKR↓GLF |
| A_Duck_Dongguan_IBC59_2021_H5N6 | Guangdong | Chicken | 2021/9/9 | H5N6 | RRKR↓GLF |

**Appendix Table 2.** A_Duck_Donggaun_IBC59_2021_H5N6 genomic similarity

| Gene | Name | Similarity | Subtype |
|---|---|---|---|
| HA | A/goose/China/21FU008/2020(H5N8) | 99.06% | H5N8 |
| NA | A/China/Original/2018(H5N6) | 98.91% | H5N6 |
| PB2 | A/Cygnus columbianus/Hubei/52/2020(H5N8) | 100.00% | H5N8 |
| PB1 | A/duck/Guangxi/293D21/2017(H1N2) | 100.00% | H1N2 |
| PA | A/Cygnus columbianus/Hubei/116/2020(H5N8) | 99.69% | H5N8 |
| NP | A/chicken/Ganzhou/GZ43/2016(H3N2) | 97.42% | H3N2 |
| NS | A/duck/Mongolia/543/2015(H4N6) | 97.90% | H4N6 |
| M | A/Cygnus columbianus/Hubei/56/2020(H5N8) | 100.00% | H5N8 |

**Appendix Table 3.** A_ENV_Dongguan_ED3_2021_H5N6 genomic similarity

| Gene | Name | Similarity | Subtype |
|---|---|---|---|
| HA | A/goose/China/21FU008/2020(H5N8) | 99.59% | H5N8 |
| NA | A/goose/Fujian/3.15_FZHX0007-C/2018(H5N6) | 97.93% | H5N6 |
| PB2 | A/duck/Guangxi/293D21/2017(H1N2) | 96.71% | H1N2 |
| PB1 | A/duck/Guangxi/293D21/2017(H1N2) | 97.42% | H1N2 |
| PA | A/wild bird /Shandong/11706/2019(H9N2) | 97.95% | H9N2 |
| NP | A/duck/Hunan/5.29_YYGK90P3-OC/2018(mixed) | 95.97% | H5N6 |
| NS | A/Duck/China/B1_NS/2018(H5N6) | 99.15% | H5N6 |
| M | A/Cygnus columbianus/Hubei/56/2020(H5N8) | 100.00% | H5N8 |

**Appendix Table 4.** A_Duck_Dongguan_IBC54_2021_H5N6 genomic similarity

| Gene | Name | Similarity | Subtype |
|---|---|---|---|
| HA | A/goose/China/21FU008/2020(H5N8) | 99.47% | H5N8 |
| NA | A/China/Original/2018(H5N6) | 98.91% | H5N6 |
| PB2 | A/Cygnus columbianus/Hubei/52/2020(H5N8) | 99.79% | H5N8 |
| PB1 | A/duck/Guangxi/293D21/2017(H1N2) | 100.00% | H1N2 |
| PA | A/Cygnus columbianus/Hubei/116/2020(H5N8) | 99.69% | H5N8 |
| NP | A/duck/Mongolia/543/2015(H4N6) | 99.04% | H4N6 |
| NS | A/chicken/Ganzhou/GZ43/2016(H3N2) | 97.42% | H3N2 |
| M | A/Cygnus columbianus/Hubei/56/2020(H5N8) | 99.81% | H5N8 |

**Appendix Table 5.** A_Goose_Dongguan_D48_2021_H5N6 genomic similarity

| Gene | Name | Similarity | Subtype |
|---|---|---|---|
| HA | A/goose/China/21FU008/2020(H5N8) | 99.59% | H5N8 |
| NA | A/duck/China/FJ1904/2019(H5N6) | 98.48% | H5N6 |
| PB2 | A/Cygnus columbianus/Hubei/52/2020(H5N8) | 99.69% | H5N8 |
| PB1 | A/duck/Guangxi/293D21/2017(H1N2) | 97.61% | H1N2 |
| PA | A/Cygnus columbianus/Hubei/116/2020(H5N8) | 99.87% | H5N8 |
| NP | A/duck/Mongolia/543/2015(H4N6) | 99.10% | H4N6 |
| NS | A/chicken/Ganzhou/GZ43/2016(H3N2) | 97.30% | H3N2 |
| M | A/Cygnus columbianus/Hubei/56/2020(H5N8) | 99.81% | H5N8 |

**Appendix Table 6.** A_Duck_Dongguan_LD9_2021_H5N6 genomic similarity

| Gene | Name | Similarity | Subtype |
|---|---|---|---|
| HA | A/Cygnus columbianus/Hubei/53/2020(H5N8) | 99.47% | H5N8 |
| NA | A/China/Original/2018(H5N6) | 98.77% | H5N6 |
| PB2 | A/Cygnus columbianus/Hubei/52/2020(H5N8) | 99.70% | H5N8 |
| PB1 | A/duck/Guangxi/293D21/2017(H1N2) | 97.65% | H1N2 |
| PA | A/Cygnus columbianus/Hubei/116/2020(H5N8) | 99.60% | H5N8 |
| NP | A/duck/Mongolia/543/2015(H4N6) | 99.16% | H4N6 |
| NS | A/duck/Zhejiang/10.26_HZBX001-C/2018(mixed) | 97.09% | H5N6 |
| M | A/Cygnus columbianus/Hubei/56/2020(H5N8) | 99.71% | H5N8 |

**Appendix Table 7.** A_Chicken_Chongqing_001_2021_H5N6 genomic similarity

| Gene | Name | Similarity | Subtype |
|---|---|---|---|
| HA | A/Cygnus columbianus/Hubei/53/2020(H5N8) | 99.18% | H5N8 |
| NA | A/Muscovy duck/China/H5N6/2020(H5N6) | 99.28% | H5N6 |
| PB2 | A/chicken/Guangxi/165C7/2014(H3N2) | 96.88% | H3N2 |
| PB1 | A/duck/Guangxi/293D21/2017(H1N2) | 97.82% | H1N2 |
| PA | A/duck/China/322D22/2018(H3N2) | 96.51% | H3N2 |
| NP | A/Muscovy duck/China/H5N6/2020(H5N6) | 95.19% | H5N6 |
| NS | A/chicken/Ganzhou/GZ43/2016(H3N2) | 97.85% | H3N2 |
| M | A/Cygnus columbianus/Hubei/56/2020(H5N8) | 99.81% | H5N8 |

**Appendix Table 8.** A_Goose_Guangdong_210520_2021_H5N6 genomic similarity

| Gene | Name | Similarity | Subtype |
|---|---|---|---|
| HA | A/Cygnus columbianus/Hubei/53/2020(H5N8) | 99.55% | H5N8 |
| NA | A/Muscovy duck/China/H5N6/2020(H5N6) | 99.13% | H5N6 |
| PB2 | A/Cygnus columbianus/Hubei/52/2020(H5N8) | 99.70% | H5N8 |
| PB1 | A/duck/Guangxi/293D21/2017(H1N2) | 97.39% | H1N2 |
| PA | A/Cygnus columbianus/Hubei/116/2020(H5N8) | 99.55% | H5N8 |
| NP | A/duck/Mongolia/543/2015(H4N6) | 99.22% | H4N6 |
| NS | A/chicken/Ganzhou/GZ43/2016(H3N2) | 97.53% | H3N2 |
| M | A/Cygnus columbianus/Hubei/56/2020(H5N8) | 99.71% | H5N8 |

**Appendix Table 9.** A_Chicken_Guangdong_210518_2021_H5N6 genomic similarity

| Gene | Name | Similarity | Subtype |
|---|---|---|---|
| HA | A/Cygnus columbianus/Hubei/53/2020(H5N8) | 99.77% | H5N8 |
| NA | A/duck/China/FJ1904/2019(H5N6) | 97.97% | H5N6 |
| PB2 | A/Duck/China/B6_PB2/2019(H5N6) | 98.33% | H5N6 |
| PB1 | A/duck/Mongolia/MN18-1/2018(H3N6) | 99.05% | H3N6 |
| PA | A/duck/Laos/XBY004/2014(H5N6) | 98.22% | H5N8 |
| NP | A/duck/Guangdong/8.30_DGCP030-C/2017(mixed) | 98.40% | H5N6 |
| NS | A/goose/Guangdong/7.20_DGCP010-C/2017(H5N6) | 98.74% | H5N6 |
| M | A/Cygnus columbianus/Hubei/56/2020(H5N8) | 99.32% | H5N8 |

**Appendix Table 10.** A_Chicken_Guangzhou_210539_2021_H5N6 genomic similarity

| Gene | Name | Similarity | Subtype |
| --- | --- | --- | --- |
| HA | A/Cygnus columbianus/Hubei/53/2020(H5N8) | 99.55% | H5N8 |
| NA | A/Muscovy duck/China/H5N6/2020(H5N6) | 99.13% | H5N6 |
| PB2 | A/chicken/Guangxi/165C7/2014(H3N2) | 96.71% | H3N2 |
| PB1 | A/duck/Guangxi/293D21/2017(H1N2) | 97.74% | H1N2 |
| PA | A/duck/China/322D22/2018(H3N2) | 97.76% | H3N2 |
| NP | A/duck/Hunan/4.26_YYGK90R3-OC/2018(mixed) | 98.66% | H5N6 |
| NS | A/chicken/Ganzhou/GZ43/2016(H3N2) | 98.09% | H3N2 |
| M | A/Cygnus columbianus/Hubei/56/2020(H5N8) | 99.71% | H5N8 |

**Appendix Table 11.** A_Chicken_Guangzhou_210539_2021_H5N6 genomic similarity

| Gene | Name | Similarity | Subtype |
| --- | --- | --- | --- |
| HA | A/goose/Guangdong/7.20_DGCP010-C/2017(H5N6) | 97.46% | H5N6 |
| NA | A/duck/Hainan/12.29_HKPL002-C/2017(H5N6) | 97.82% | H5N6 |
| PB2 | A/chicken/Qingyuan/zd201601/2016(H9N2) | 98.85% | H9N2 |
| PB1 | A/Muscovy duck/Vietnam/HN4856/2018(H5N6) | 97.82% | H5N6 |
| PA | A/goose/Fujian/3.15_FZHX0007-O/2018(H5N6) | 97.98% | H5N6 |
| NP | A/goose/Fujian/3.15_FZHX0010-O/2018(H5N6) | 98.78% | H5N6 |
| NS | A/goose/Guangdong/7.20_DGCP010-C/2017(H5N6) | 98.40% | H5N6 |
| M | A/Muscovy duck/Vietnam/HN5135/2018(H5N6) | 99.42% | H5N6 |

**Appendix Table 12.** Hemagglutinin inhibition tests of antigen differences between influenza A (H5N6) virus and vaccine in China in 2021

| | Antibody | | | |
|---|---|---|---|---|
| Antigen | Re-11 | Re-12 | rFJ56 | rSD57 |
| A_Chicken_Chongqing_001_2021_H5N6 | 2 | 0 | 2 | 0 |
| A_Goose_Guangdong_210520_2021_H5N6 | 2 | 0 | 2 | 0 |
| A_Chicken_Guangxi_210539_2021_H5N6 | 2 | 0 | 2 | 0 |
| A_Goose_Dongguan_D48_2021_H5N6 | 5 | 3 | 3 | 2 |
| A_Duck_Dongguan_LD9_2021_H5N6 | 3 | 2 | 3 | 0 |
| A_ENV_Dongguan_ED3_2021_H5N6 | 0 | 0 | 0 | 0 |
| A_Duck_Dongguan_IBC54_2021_H5N6 | 1 | 0 | 0 | 0 |
| A_Duck_Dongguan_IBC59_2021_H5N6 | 3 | 0 | 3 | 1 |
| Re-11 | 8 | - | - | - |
| Re-12 | - | 9 | - | - |
| rFJ56 | - | - | 10 | - |
| rSD57 | - | - | - | 9 |

Re-11, Re-12, rFJ56 and rSD57 are vaccine strains widely used in China. Both antigen and antiserum of Re-11 and Re-12 were purchased from the Harbin Weike Biotechnology Development Company (www.hvriwk.com). Both antigen and antiserum of rFJ56 and rSD57 were from Guangzhou South China Biologic Medicine (http://www.gzscbm.com).

**Appendix Table 13.** A_Chongqing_00013_202_H5N6 genomic similarity

| Gene | Name | Similarity | Subtype |
|---|---|---|---|
| HA | A/goose/Guangdong/7.20_DGCP010-C/2017(H5N6) | 97.46% | H5N6 |
| NA | A/duck/Hainan/12.29_HKPL002-C/2017(H5N6) | 97.82% | H5N6 |
| PB2 | A/chicken/Qingyuan/zd201601/2016(H9N2) | 98.85% | H9N2 |
| PB1 | A/Muscovy duck/Vietnam/HN4856/2018(H5N6) | 97.82% | H5N6 |
| PA | A/goose/Fujian/3.15_FZHX0007-O/2018(H5N6) | 97.98% | H5N6 |
| NP | A/goose/Fujian/3.15_FZHX0010-O/2018(H5N6) | 98.78% | H5N6 |
| NS | A/goose/Guangdong/7.20_DGCP010-C/2017(H5N6) | 98.40% | H5N6 |
| M | A/Muscovy duck/Vietnam/HN5135/2018(H5N6) | 99.42% | H5N6 |

**Appendix Table 14.** A_GX-guilin_11151_202_H5N6 genomic similarity

| Gene | Name | Similarity | Subtype |
|---|---|---|---|
| HA | A/Cygnus columbianus/Hubei/53/2020(H5N8) | 99.66% | H5N8 |
| NA | A/Muscovy duck/Vietnam/HN6606/2020(H5N6) | 99.02% | H5N6 |
| PB2 | A/duck/Guangxi/293D21/2017(H1N2) | 96.50% | H1N2 |
| PB1 | A/duck/Guangxi/293D21/2017(H1N2) | 97.18% | H1N2 |
| PA | A/duck/Vietnam/HN5958/2019(H3N8) | 97.58% | H3N8 |
| NP | A/chicken/Shandong/2.28_TAWM016-C/2017(H5N6) | 98.72% | H5N6 |
| NS | A/Muscovy duck/Vietnam/HN6610/2020(H5N6) | 99.54% | H5N6 |
| M | A/Cygnus columbianus/Hubei/56/2020(H5N8) | 99.61% | H5N8 |

**Appendix Table 15.** A_Hangzhou_01_202_H5N6 genomic similarity

| Gene | Name | Similarity | Subtype |
|---|---|---|---|
| HA | A/Cygnuscolumbianus/Hubei/53/2020(H5N8) | 99.16% | H5N8 |
| NA | A/Muscovy duck/Vietnam/HN6606/2020(H5N6) | 98.77% | H5N6 |
| PB2 | A/duck/Guangxi/293D21/2017(H1N2) | 96.53% | H1N2 |
| PB1 | A/duck/Guangxi/293D21/2017(H1N2) | 97.05% | H1N2 |
| PA | A/wild bird /Shandong/11706/2019(H9N2) | 97.77% | H9N2 |
| NP | A/chicken/Anhui/8.28_YHZGS017-O/2018(H5N6) | 98.19% | H5N6 |
| NS | A/Muscovy duck/Vietnam/HN6609/2020(H5N6) | 99.03% | H5N6 |
| M | A/eurasian coot/Shandong/W5611/2020(H5N8) | 99.49% | H5N8 |

**Appendix Table 16.** A_Sichuan_06681_2021_H5N6 genomic similarity

| Gene | Name | Similarity | Subtype |
|---|---|---|---|
| HA | A/environment sample/China/TZ001/2021(H5N8) | 99.44% | H5N8 |
| NA | A/duck/Hainan/12.29_HKPL002-C/2017(H5N6) | 97.54% | H5N6 |
| PB2 | A/chicken/Guangxi/165C7/2014(H3N2) | 96.63% | H3N2 |
| PB1 | A/duck/Guangxi/293D21/2017(H1N2) | 97.86% | H1N2 |
| PA | A/duck/China/322D22/2018(H3N2) | 97.76% | H3N2 |
| NP | A/duck/Hunan/4.26_YYGK90R3-OC/2018(mixed) | 98.66% | H5N6 |
| NS | A/chicken/Ganzhou/GZ43/2016(H3N2) | 98.09% | H3N2 |
| M | A/Cygnus columbianus/Hubei/56/2020(H5N8) | 99.90% | H5N8 |

**Appendix Table 17.** A_GX_hechi_01_2021_H5N6 genomic similarity

| Gene | Name | Similarity | Subtype |
|---|---|---|---|
| HA | A/environment/Anhui/11.29_YHZGS003-E/2018 | 97.74% | H5N8 |
| NA | A/Muscovy duck/Vietnam/HN6606/2020(H5N6) | 99.23% | H5N6 |
| PB2 | A/goose/Fujian/3.15_FZHX0011-O/2018(H9N2) | 92.18% | H9N2 |
| PB1 | A/duck/China/322D22/2018(H3N2) | 94.79% | H3N2 |
| PA | A/duck/Vietnam/HN5958/2019(H3N8) | 97.04% | H3N8 |
| NP | A/duck/Vietnam/HN6611/2020(H5N6) | 99.62% | H5N6 |
| NS | A/Muscovy duck/Vietnam/HN6610/2020(H5N6) | 99.43% | H5N6 |
| M | A/chicken/Hunan/4.26_YYGK37R3-OC/2018(mixed) | 98.64% | H5N8 |

**Appendix Table 18.** The accession numbers in GISAID of the influenza A(H5N6) viruses in this study

| Name | ID sequence | Time |
|---|---|---|
| A_Duck_Chengdu_210462_2021_H5N6 | EPI_ISL_5797454 | 2021/6/30 |
| A_Chicken_Chongqing_001_2021_H5N6 | EPI_ISL_5797453 | 2021/7/29 |
| A_Goose_Guangdong_210520_2021_H5N6 | EPI_ISL_5797280 | 2021/8/24 |
| A_Chicken_Guangdong_210518_2021_H5N6 | EPI_ISL_5797279 | 2021/8/24 |
| A_Chicken_Guangxi_210539_2021_H5N6 | EPI_ISL_5797272 | 2021/9/23 |
| A_Goose_Dongguan_D48_2021_H5N6 | EPI_ISL_5797238 | 2021/9/2 |
| A_Duck_Dongguan_LD9_2021_H5N6 | EPI_ISL_5797237 | 2021/9/15 |
| A_ENV_Dongguan_ED3_2021_H5N6 | EPI_ISL_5797236 | 2021/9/1 |
| A_Duck_Dongguan_IBC54_2021_H5N6 | EPI_ISL_5797235 | 2021/9/7 |
| A_Duck_Dongguan_IBC59_2021_H5N6 | EPI_ISL_5795871 | 2021/9/9 |

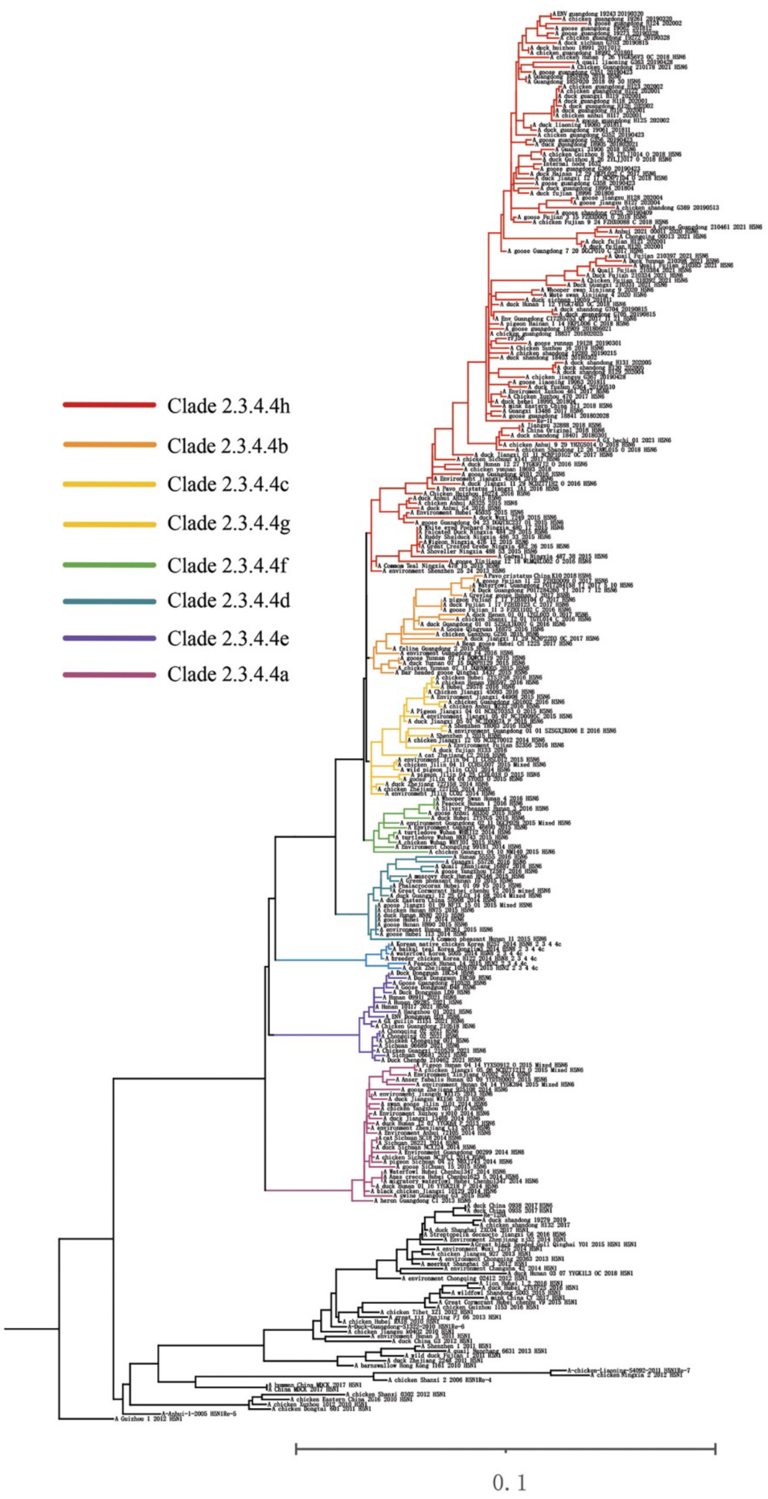


**Appendix Figure 1.** Phylogenic tree of HA gene sequences. The total HA genes (n=342) of H5 viruses collected from 2000-2021 in China were analyzed. The tree is rooted to A/Guizhou/1/2012/H5N6. The red color indicates the novel H5N6 isolates in this study.

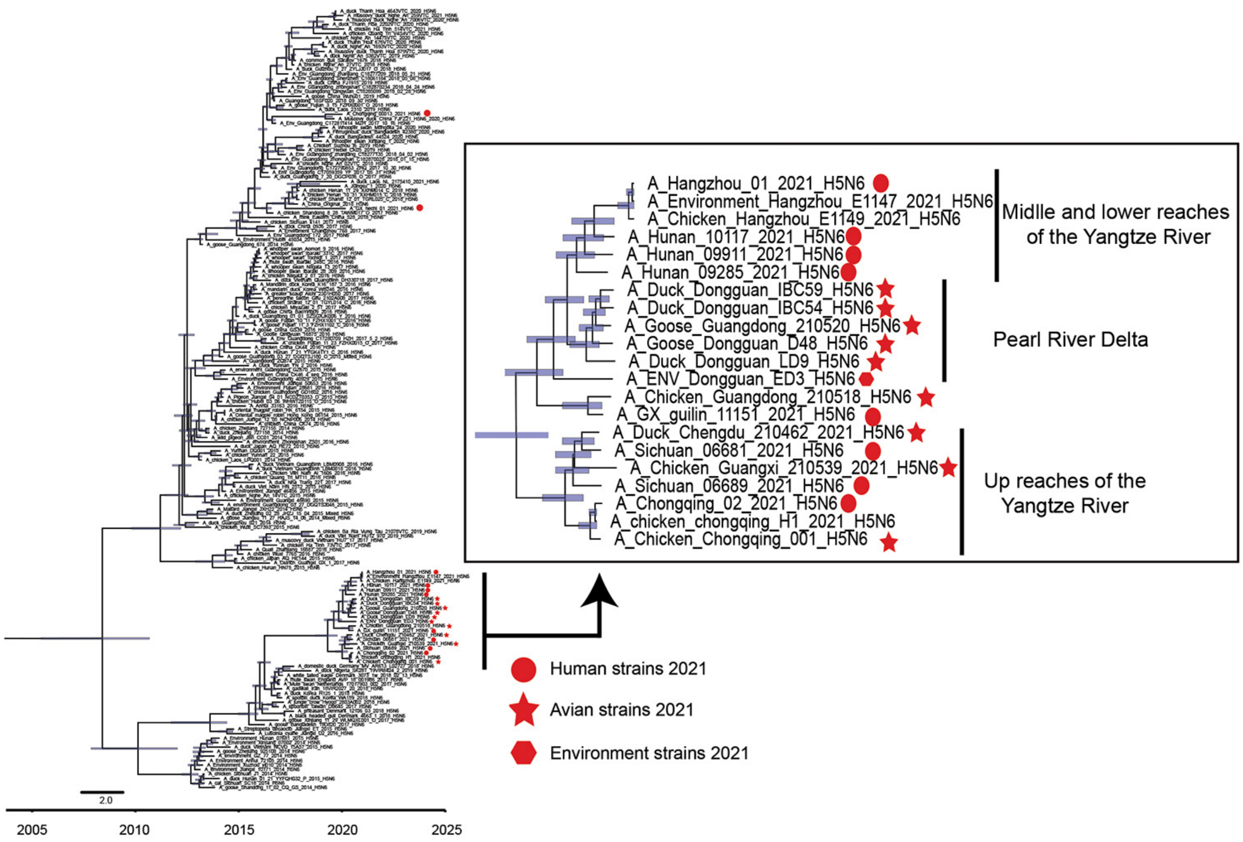


**Appendix Figure 2.** Maximum confidence tree of the HA gene sequence. The total number of HA genes (n = 174) from H5N6 viruses collected in 2014-2021 was analyzed. The red circle represents the human origin H5N6 isolate in this study, and the Red Pentagram represents the avian origin isolate in this study. The MCMC chain was run for 100 million steps with tree sampling every 1000 steps, and a 10% burn-in fraction was used when calculating the final MCC tree with common ancestor node heights in TreeAnnotator v.1.10.4.

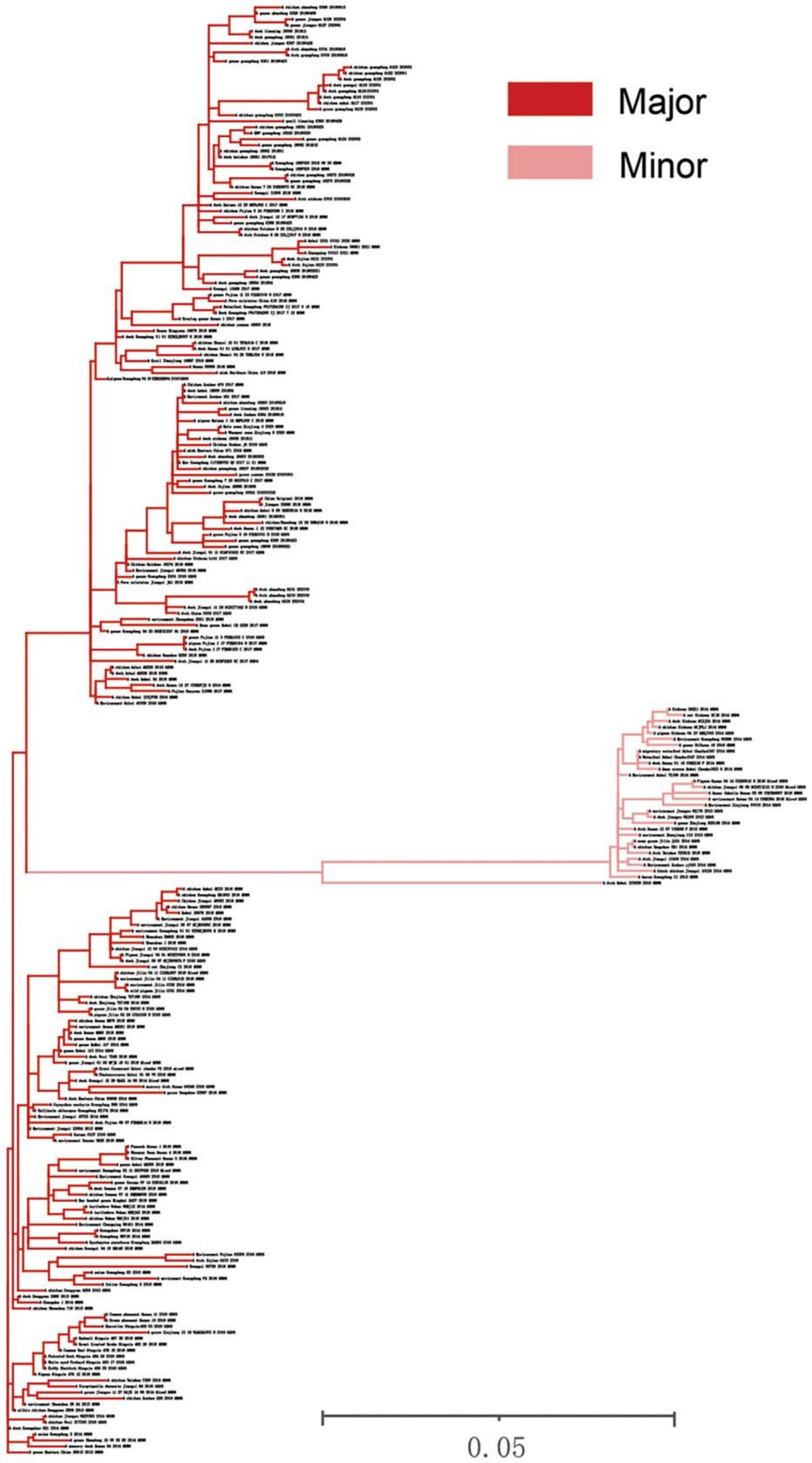


**Appendix Figure 3.** Phylogenic tree of NA gene sequences. The total NA genes (n=242) of H5 viruses collected from 2000-2021 in China were analyzed.

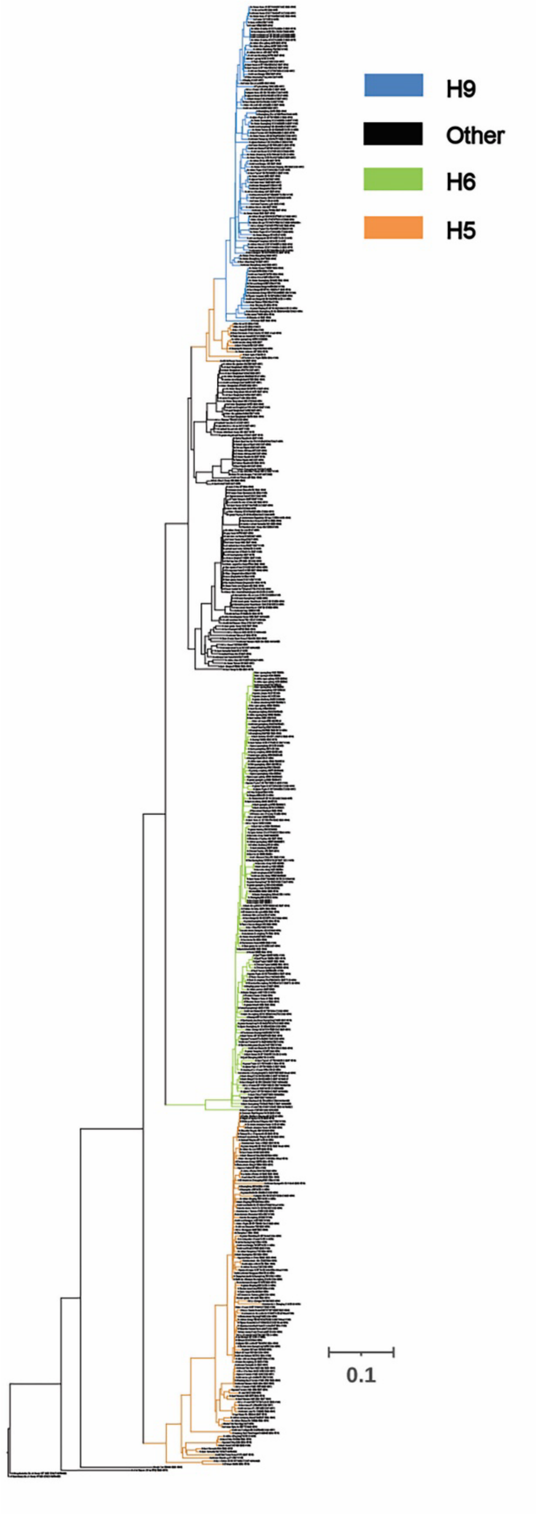


**Appendix Figure 4.** Phylogenic tree of PB2 gene sequences. The phylogenetic tree was drawn with the multiple subtype sequences possible for H5N6, which were considered to be highly similar to the subtype when it was in the same branch as the corresponding other subtype sequences.

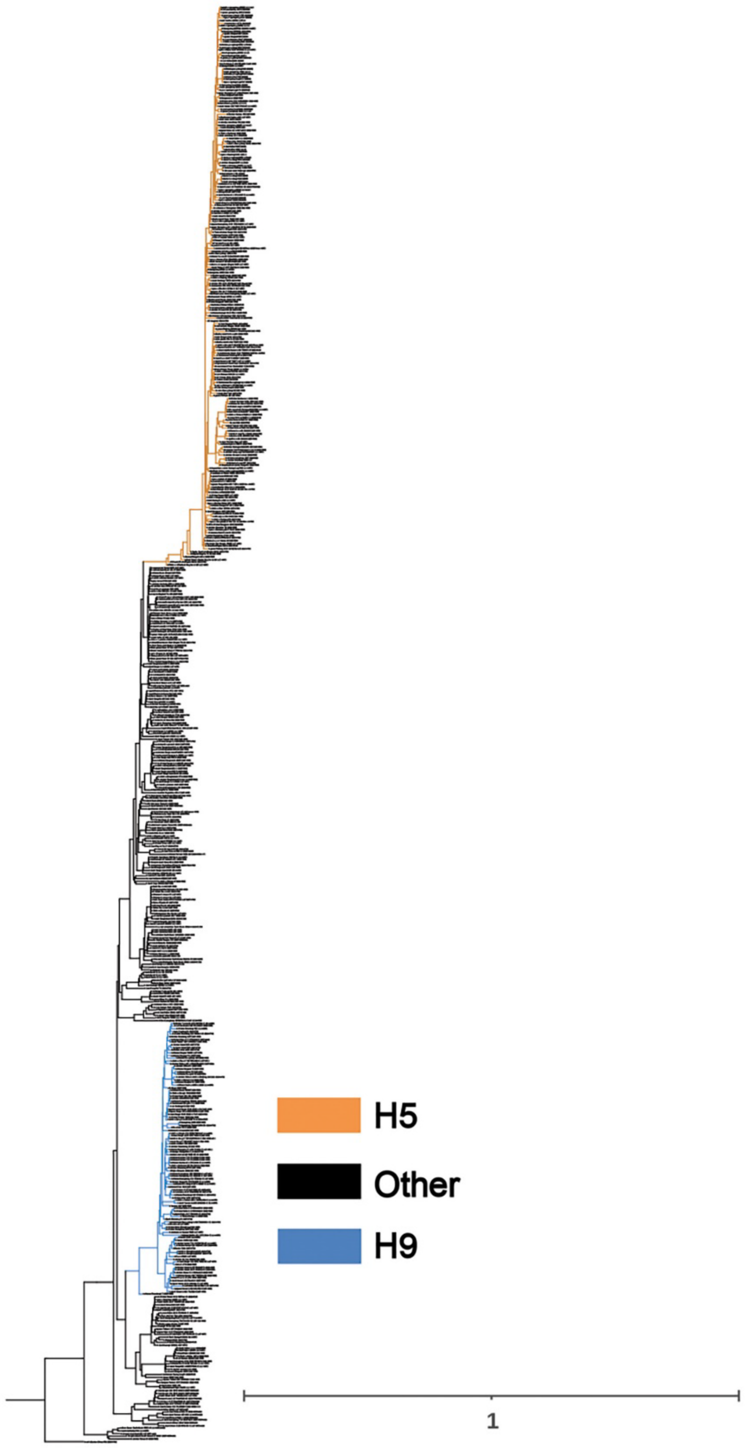


**Appendix Figure 5.** Phylogenic tree of PB1 gene sequences. The phylogenetic tree was drawn with the multiple subtype sequences possible for H5N6, which were considered to be highly similar to the subtype when it was in the same branch as the corresponding other subtype sequences.

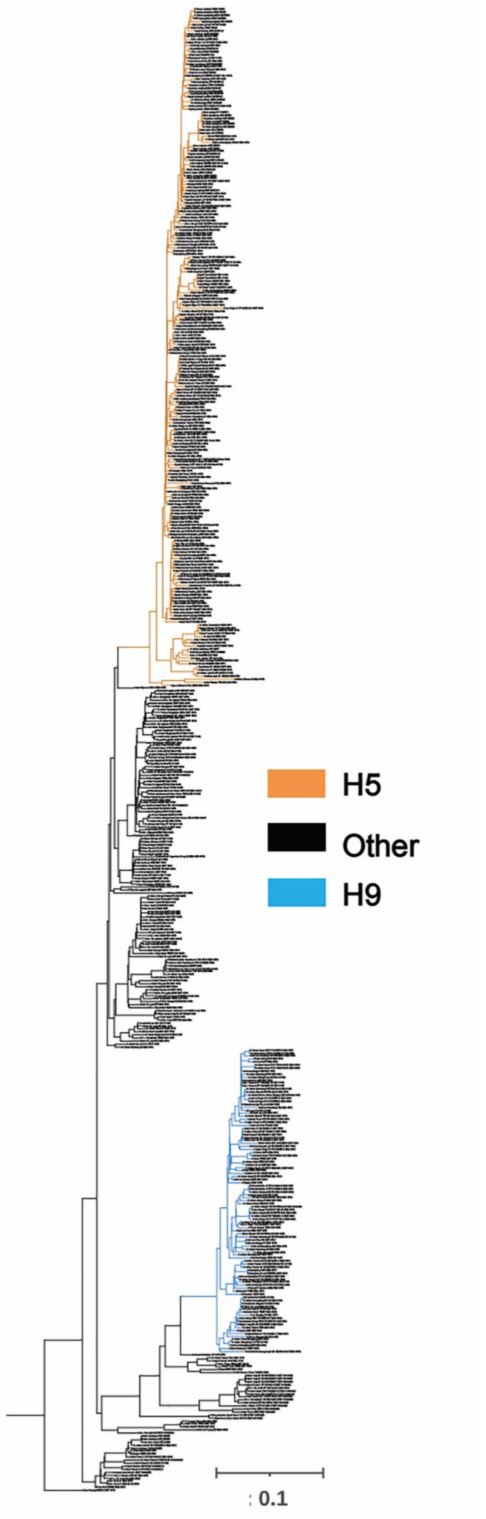


**Appendix Figure 6.** Phylogenic tree of PA gene sequences. The phylogenetic tree was drawn with the multiple subtype sequences possible for H5N6, which were considered to be highly similar to the subtype when it was in the same branch as the corresponding other subtype sequences.

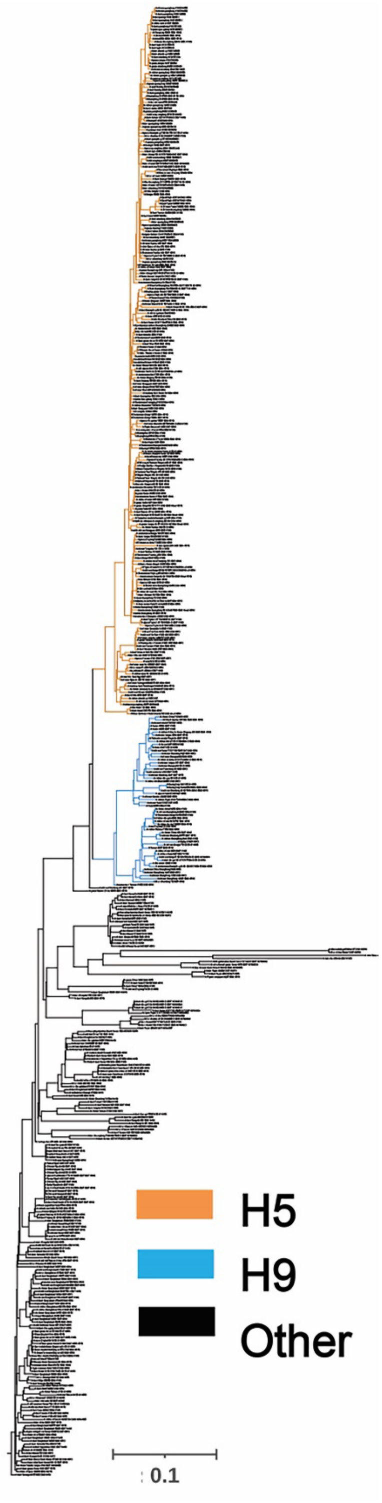


**Appendix Figure 7.** Phylogenic tree of NP gene sequences. The phylogenetic tree was drawn with the multiple subtype sequences possible for H5N6, which were considered to be highly similar to the subtype when it was in the same branch as the corresponding other subtype sequences.

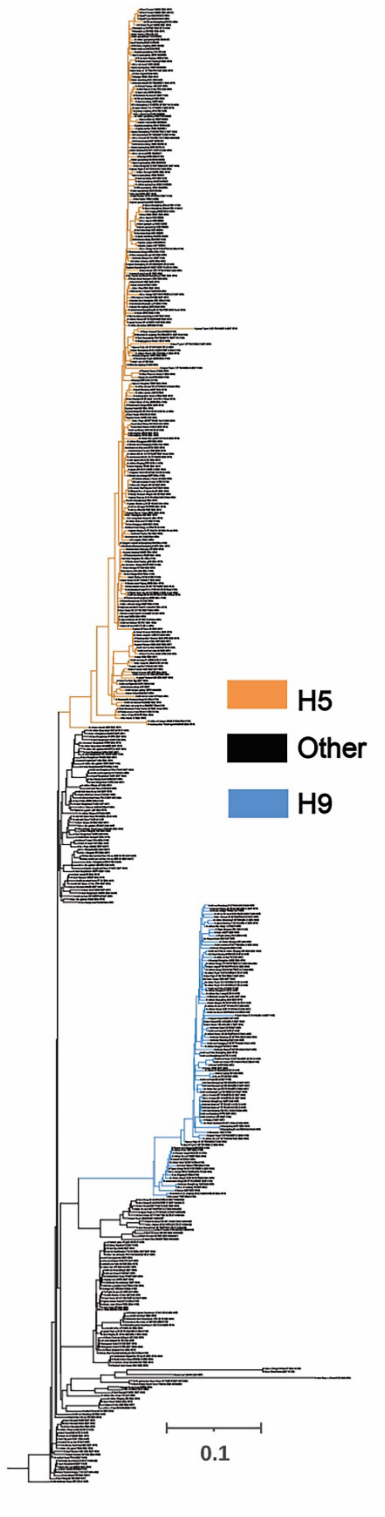


**Appendix Figure 8.** Phylogenic tree of NS gene sequences. The phylogenetic tree was drawn with the multiple subtype sequences possible for H5N6, which were considered to be highly similar to the subtype when it was in the same branch as the corresponding other subtype sequences.

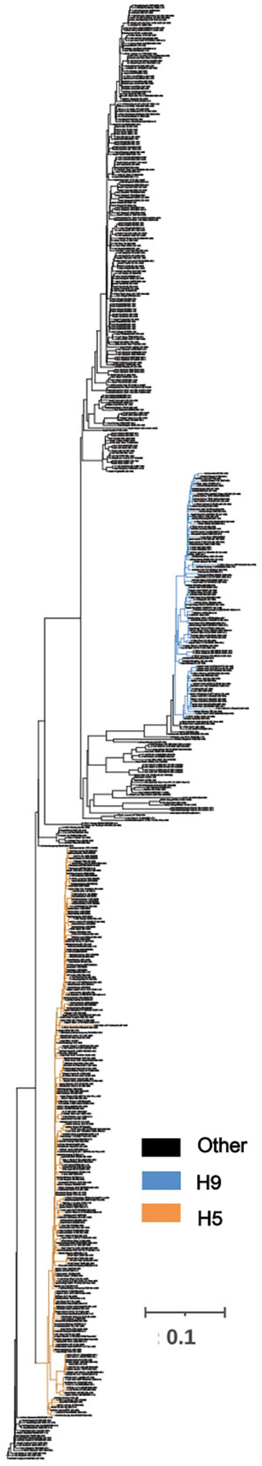


**Appendix Figure 9.** Phylogenic tree of M gene sequences. The phylogenetic tree was drawn with the multiple subtype sequences possible for H5N6, which were considered to be highly similar to the subtype when it was in the same branch as the corresponding other subtype sequences.
